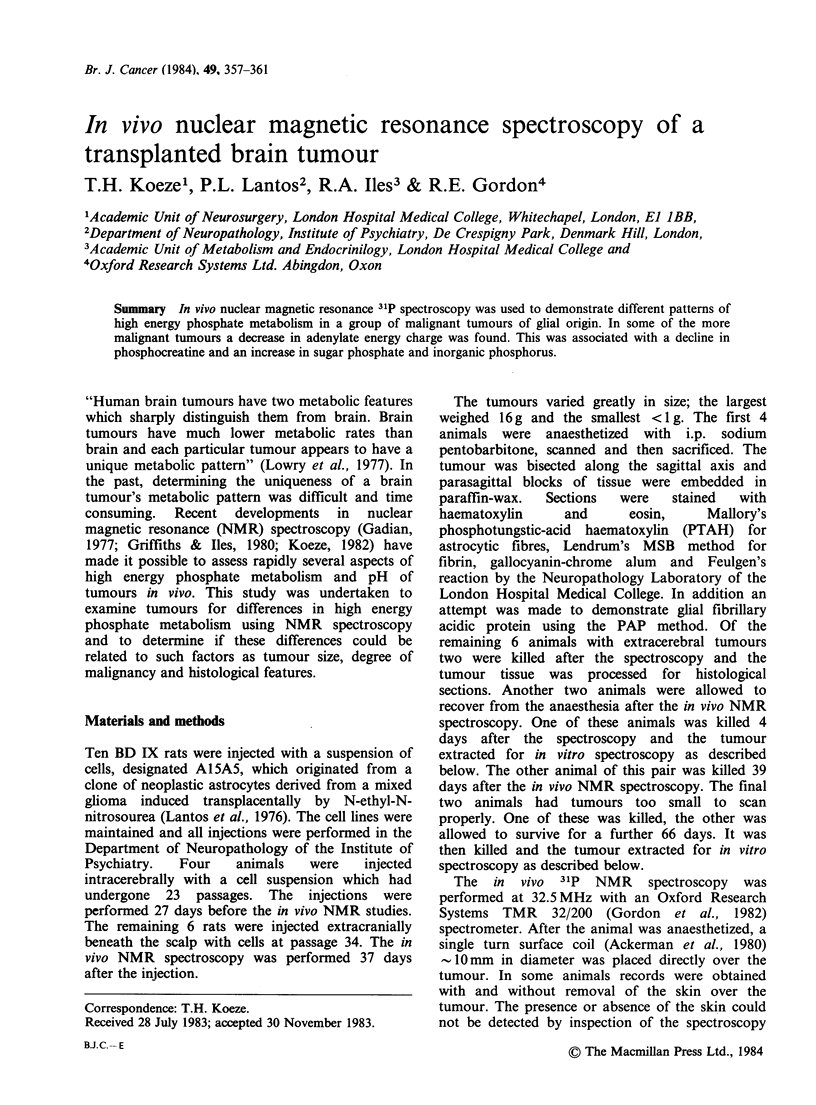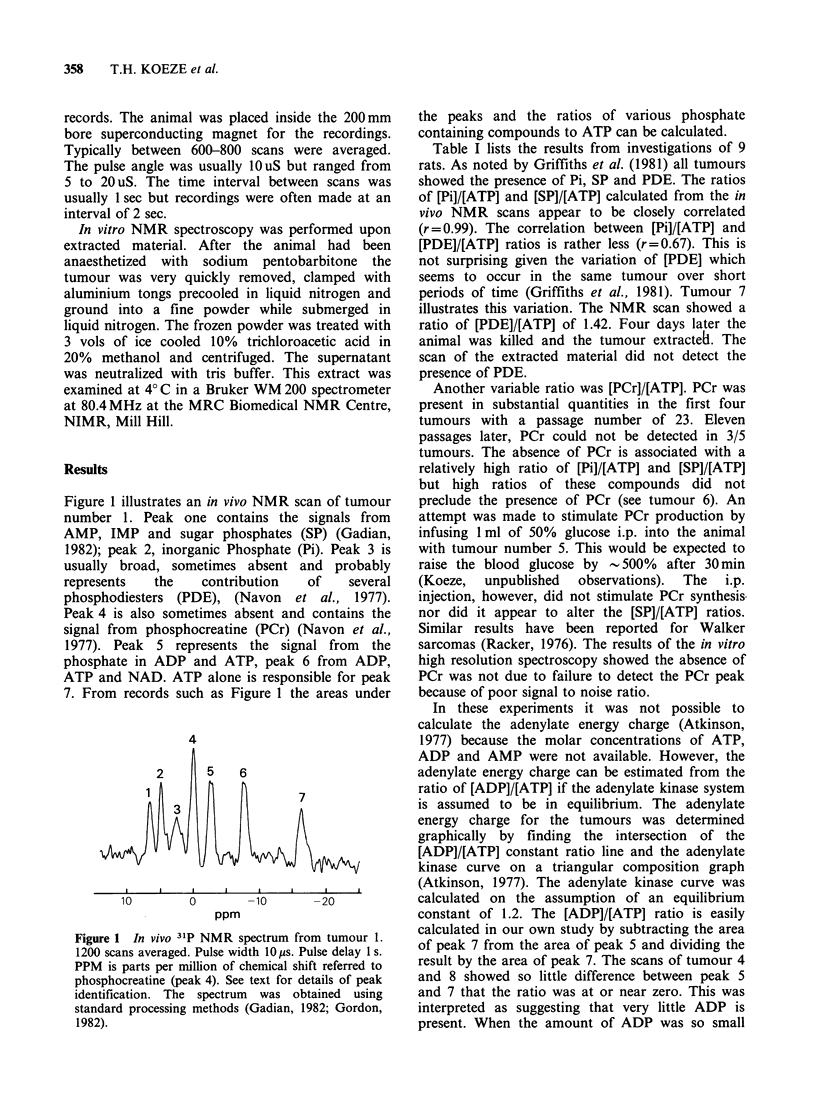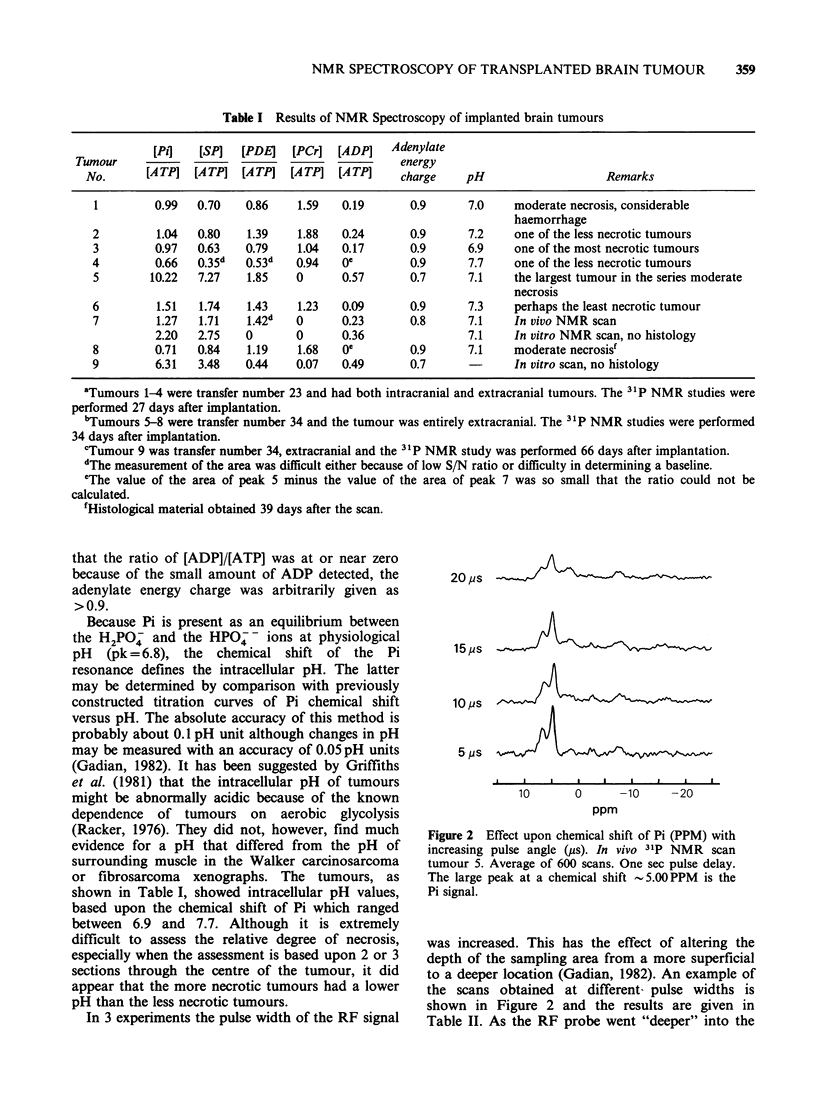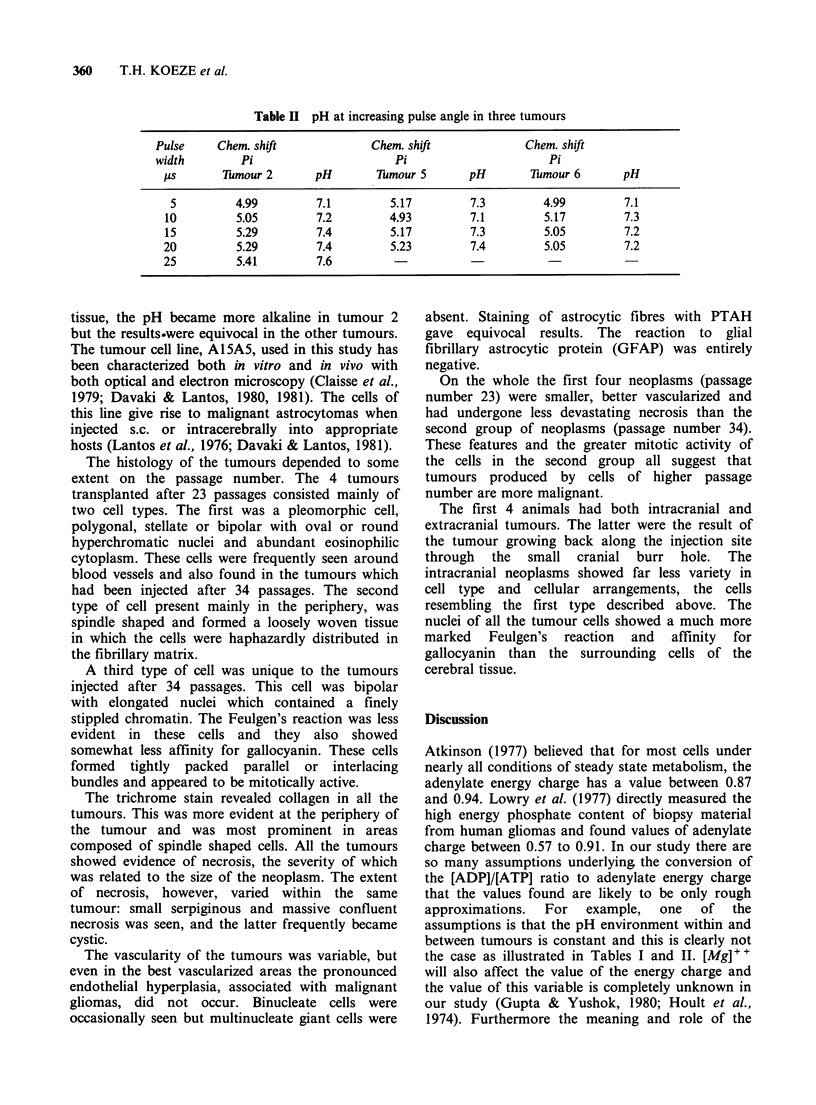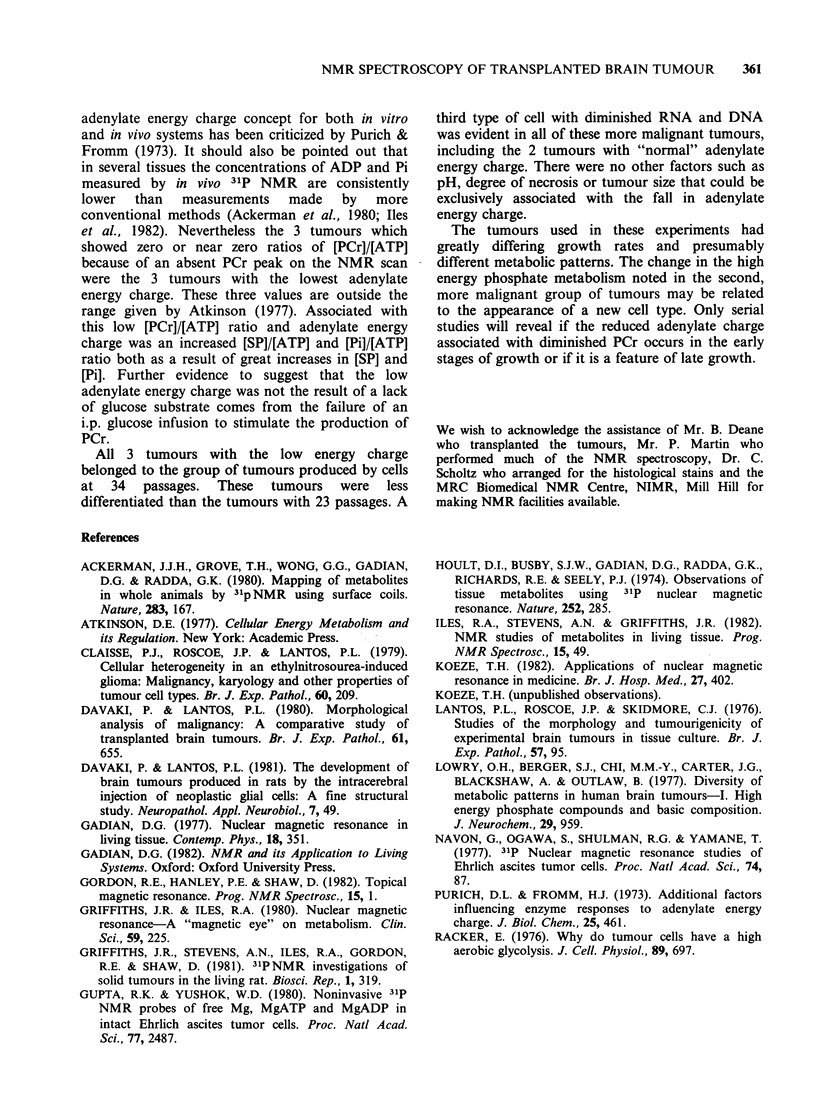# In vivo nuclear magnetic resonance spectroscopy of a transplanted brain tumour.

**DOI:** 10.1038/bjc.1984.56

**Published:** 1984-03

**Authors:** T. H. Koeze, P. L. Lantos, R. A. Iles, R. E. Gordon

## Abstract

In vivo nuclear magnetic resonance 31P spectroscopy was used to demonstrate different patterns of high energy phosphate metabolism in a group of malignant tumours of glial origin. In some of the more malignant tumours a decrease in adenylate energy charge was found. This was associated with a decline in phosphocreatine and an increase in sugar phosphate and inorganic phosphorus.


*Br. J. Cancer* (1984), **49**, 357–361

### *In vivo* nuclear magnetic resonance spectroscopy of a transplanted brain tumour

T.H. Koeze[1], P.L. Lantos[2], R.A. Iles[3] & R.E. Gordon[4]

[1]*Academic Unit of Neurosurgery, London Hospital Medical College, Whitechapel, London, E1 1BB,*
[2]*Department of Neuropathology, Institute of Psychiatry, De Crespigny Park, Denmark Hill, London,*
[3]*Academic Unit of Metabolism and Endocrinilogy, London Hospital Medical College and*
[4]*Oxford Research Systems Ltd. Abingdon, Oxon*

**Summary** *In vivo* nuclear magnetic resonance $^{31}$P spectroscopy was used to demonstrate different patterns of high energy phosphate metabolism in a group of malignant tumours of glial origin. In some of the more malignant tumours a decrease in adenylate energy charge was found. This was associated with a decline in phosphocreatine and an increase in sugar phosphate and inorganic phosphorus.

"Human brain tumours have two metabolic features which sharply distinguish them from brain. Brain tumours have much lower metabolic rates than brain and each particular tumour appears to have a unique metabolic pattern" (Lowry *et al.*, 1977). In the past, determining the uniqueness of a brain tumour's metabolic pattern was difficult and time consuming. Recent developments in nuclear magnetic resonance (NMR) spectroscopy (Gadian, 1977; Griffiths & Iles, 1980; Koeze, 1982) have made it possible to assess rapidly several aspects of high energy phosphate metabolism and pH of tumours *in vivo*. This study was undertaken to examine tumours for differences in high energy phosphate metabolism using NMR spectroscopy and to determine if these differences could be related to such factors as tumour size, degree of malignancy and histological features.

### Materials and methods

Ten BD IX rats were injected with a suspension of cells, designated A15A5, which originated from a clone of neoplastic astrocytes derived from a mixed glioma induced transplacentally by N-ethyl-N-nitrosourea (Lantos *et al.*, 1976). The cell lines were maintained and all injections were performed in the Department of Neuropathology of the Institute of Psychiatry. Four animals were injected intracerebrally with a cell suspension which had undergone 23 passages. The injections were performed 27 days before the *in vivo* NMR studies. The remaining 6 rats were injected extracranially beneath the scalp with cells at passage 34. The *in vivo* NMR spectroscopy was performed 37 days after the injection.

Correspondence: T.H. Koeze.
Received 28 July 1983; accepted 30 November 1983.

The tumours varied greatly in size; the largest weighed 16 g and the smallest <1 g. The first 4 animals were anaesthetized with i.p. sodium pentobarbitone, scanned and then sacrificed. The tumour was bisected along the sagittal axis and parasagittal blocks of tissue were embedded in paraffin-wax. Sections were stained with haematoxylin and eosin, Mallory's phosphotungstic-acid haematoxylin (PTAH) for astrocytic fibres, Lendrum's MSB method for fibrin, gallocyanin-chrome alum and Feulgen's reaction by the Neuropathology Laboratory of the London Hospital Medical College. In addition an attempt was made to demonstrate glial fibrillary acidic protein using the PAP method. Of the remaining 6 animals with extracerebral tumours two were killed after the spectroscopy and the tumour tissue was processed for histological sections. Another two animals were allowed to recover from the anaesthesia after the *in vivo* NMR spectroscopy. One of these animals was killed 4 days after the spectroscopy and the tumour extracted for *in vitro* spectroscopy as described below. The other animal of this pair was killed 39 days after the *in vivo* NMR spectroscopy. The final two animals had tumours too small to scan properly. One of these was killed, the other was allowed to survive for a further 66 days. It was then killed and the tumour extracted for *in vitro* spectroscopy as described below.

The *in vivo* $^{31}$P NMR spectroscopy was performed at 32.5 MHz with an Oxford Research Systems TMR 32/200 (Gordon *et al.*, 1982) spectrometer. After the animal was anaesthetized, a single turn surface coil (Ackerman *et al.*, 1980) ~10 mm in diameter was placed directly over the tumour. In some animals records were obtained with and without removal of the skin over the tumour. The presence or absence of the skin could not be detected by inspection of the spectroscopy

© The Macmillan Press Ltd., 1984

records. The animal was placed inside the 200 mm bore superconducting magnet for the recordings. Typically between 600–800 scans were averaged. The pulse angle was usually 10 uS but ranged from 5 to 20 uS. The time interval between scans was usually 1 sec but recordings were often made at an interval of 2 sec.

*In vitro* NMR spectroscopy was performed upon extracted material. After the animal had been anaesthetized with sodium pentobarbitone the tumour was very quickly removed, clamped with aluminium tongs precooled in liquid nitrogen and ground into a fine powder while submerged in liquid nitrogen. The frozen powder was treated with 3 vols of ice cooled 10% trichloroacetic acid in 20% methanol and centrifuged. The supernatant was neutralized with tris buffer. This extract was examined at 4° C in a Bruker WM 200 spectrometer at 80.4 MHz at the MRC Biomedical NMR Centre, NIMR, Mill Hill.

### Results

Figure 1 illustrates an *in vivo* NMR scan of tumour number 1. Peak one contains the signals from AMP, IMP and sugar phosphates (SP) (Gadian, 1982); peak 2, inorganic Phosphate (Pi). Peak 3 is usually broad, sometimes absent and probably represents the contribution of several phosphodiesters (PDE), (Navon *et al.*, 1977). Peak 4 is also sometimes absent and contains the signal from phosphocreatine (PCr) (Navon *et al.*, 1977). Peak 5 represents the signal from the phosphate in ADP and ATP, peak 6 from ADP, ATP and NAD. ATP alone is responsible for peak 7. From records such as Figure 1 the areas under

**Figure 1** *In vivo* $^{31}P$ NMR spectrum from tumour 1. 1200 scans averaged. Pulse width 10 $\mu$s. Pulse delay 1 s. PPM is parts per million of chemical shift referred to phosphocreatine (peak 4). See text for details of peak identification. The spectrum was obtained using standard processing methods (Gadian, 1982; Gordon, 1982).

the peaks and the ratios of various phosphate containing compounds to ATP can be calculated.

Table I lists the results from investigations of 9 rats. As noted by Griffiths *et al.* (1981) all tumours showed the presence of Pi, SP and PDE. The ratios of [Pi]/[ATP] and [SP]/[ATP] calculated from the *in vivo* NMR scans appear to be closely correlated ($r = 0.99$). The correlation between [Pi]/[ATP] and [PDE]/[ATP] ratios is rather less ($r = 0.67$). This is not surprising given the variation of [PDE] which seems to occur in the same tumour over short periods of time (Griffiths *et al.*, 1981). Tumour 7 illustrates this variation. The NMR scan showed a ratio of [PDE]/[ATP] of 1.42. Four days later the animal was killed and the tumour extracted. The scan of the extracted material did not detect the presence of PDE.

Another variable ratio was [PCr]/[ATP]. PCr was present in substantial quantities in the first four tumours with a passage number of 23. Eleven passages later, PCr could not be detected in 3/5 tumours. The absence of PCr is associated with a relatively high ratio of [Pi]/[ATP] and [SP]/[ATP] but high ratios of these compounds did not preclude the presence of PCr (see tumour 6). An attempt was made to stimulate PCr production by infusing 1 ml of 50% glucose i.p. into the animal with tumour number 5. This would be expected to raise the blood glucose by ~500% after 30 min (Koeze, unpublished observations). The i.p. injection, however, did not stimulate PCr synthesis nor did it appear to alter the [SP]/[ATP] ratios. Similar results have been reported for Walker sarcomas (Racker, 1976). The results of the *in vitro* high resolution spectroscopy showed the absence of PCr was not due to failure to detect the PCr peak because of poor signal to noise ratio.

In these experiments it was not possible to calculate the adenylate energy charge (Atkinson, 1977) because the molar concentrations of ATP, ADP and AMP were not available. However, the adenylate energy charge can be estimated from the ratio of [ADP]/[ATP] if the adenylate kinase system is assumed to be in equilibrium. The adenylate energy charge for the tumours was determined graphically by finding the intersection of the [ADP]/[ATP] constant ratio line and the adenylate kinase curve on a triangular composition graph (Atkinson, 1977). The adenylate kinase curve was calculated on the assumption of an equilibrium constant of 1.2. The [ADP]/[ATP] ratio is easily calculated in our own study by subtracting the area of peak 7 from the area of peak 5 and dividing the result by the area of peak 7. The scans of tumour 4 and 8 showed so little difference between peak 5 and 7 that the ratio was at or near zero. This was interpreted as suggesting that very little ADP is present. When the amount of ADP was so small

**Table I** Results of NMR Spectroscopy of implanted brain tumours

| *Tumour No.* | *[Pi]/[ATP]* | *[SP]/[ATP]* | *[PDE]/[ATP]* | *[PCr]/[ATP]* | *[ADP]/[ATP]* | *Adenylate energy charge* | *pH* | *Remarks* |
|---|---|---|---|---|---|---|---|---|
| 1 | 0.99 | 0.70 | 0.86 | 1.59 | 0.19 | 0.9 | 7.0 | moderate necrosis, considerable haemorrhage |
| 2 | 1.04 | 0.80 | 1.39 | 1.88 | 0.24 | 0.9 | 7.2 | one of the less necrotic tumours |
| 3 | 0.97 | 0.63 | 0.79 | 1.04 | 0.17 | 0.9 | 6.9 | one of the most necrotic tumours |
| 4 | 0.66 | 0.35[d] | 0.53[d] | 0.94 | 0[e] | 0.9 | 7.7 | one of the less necrotic tumours |
| 5 | 10.22 | 7.27 | 1.85 | 0 | 0.57 | 0.7 | 7.1 | the largest tumour in the series moderate necrosis |
| 6 | 1.51 | 1.74 | 1.43 | 1.23 | 0.09 | 0.9 | 7.3 | perhaps the least necrotic tumour |
| 7 | 1.27 | 1.71 | 1.42[d] | 0 | 0.23 | 0.8 | 7.1 | *In vivo* NMR scan |
| | 2.20 | 2.75 | 0 | 0 | 0.36 | | 7.1 | *In vitro* NMR scan, no histology |
| 8 | 0.71 | 0.84 | 1.19 | 1.68 | 0[e] | 0.9 | 7.1 | moderate necrosis[f] |
| 9 | 6.31 | 3.48 | 0.44 | 0.07 | 0.49 | 0.7 | — | *In vitro* scan, no histology |

[a]Tumours 1–4 were transfer number 23 and had both intracranial and extracranial tumours. The $^{31}P$ NMR studies were performed 27 days after implantation.

[b]Tumours 5–8 were transfer number 34 and the tumour was entirely extracranial. The $^{31}P$ NMR studies were performed 34 days after implantation.

[c]Tumour 9 was transfer number 34, extracranial and the $^{31}P$ NMR study was performed 66 days after implantation.

[d]The measurement of the area was difficult either because of low S/N ratio or difficulty in determining a baseline.

[e]The value of the area of peak 5 minus the value of the area of peak 7 was so small that the ratio could not be calculated.

[f]Histological material obtained 39 days after the scan.

that the ratio of [ADP]/[ATP] was at or near zero because of the small amount of ADP detected, the adenylate energy charge was arbitrarily given as >0.9.

Because Pi is present as an equilibrium between the $H_2PO_4^-$ and the $HPO_4^{--}$ ions at physiological pH (pk=6.8), the chemical shift of the Pi resonance defines the intracellular pH. The latter may be determined by comparison with previously constructed titration curves of Pi chemical shift versus pH. The absolute accuracy of this method is probably about 0.1 pH unit although changes in pH may be measured with an accuracy of 0.05 pH units (Gadian, 1982). It has been suggested by Griffiths *et al.* (1981) that the intracellular pH of tumours might be abnormally acidic because of the known dependence of tumours on aerobic glycolysis (Racker, 1976). They did not, however, find much evidence for a pH that differed from the pH of surrounding muscle in the Walker carcinosarcoma or fibrosarcoma xenographs. The tumours, as shown in Table I, showed intracellular pH values, based upon the chemical shift of Pi which ranged between 6.9 and 7.7. Although it is extremely difficult to assess the relative degree of necrosis, especially when the assessment is based upon 2 or 3 sections through the centre of the tumour, it did appear that the more necrotic tumours had a lower pH than the less necrotic tumours.

In 3 experiments the pulse width of the RF signal was increased. This has the effect of altering the depth of the sampling area from a more superficial to a deeper location (Gadian, 1982). An example of the scans obtained at different pulse widths is shown in Figure 2 and the results are given in Table II. As the RF probe went "deeper" into the

**Figure 2** Effect upon chemical shift of Pi (PPM) with increasing pulse angle (μs). *In vivo* $^{31}P$ NMR scan tumour 5. Average of 600 scans. One sec pulse delay. The large peak at a chemical shift ~5.00 PPM is the Pi signal.

**Table II** pH at increasing pulse angle in three tumours

| *Pulse width µs* | *Chem. shift Pi Tumour 2* | *pH* | *Chem. shift Pi Tumour 5* | *pH* | *Chem. shift Pi Tumour 6* | *pH* |
|---|---|---|---|---|---|---|
| 5 | 4.99 | 7.1 | 5.17 | 7.3 | 4.99 | 7.1 |
| 10 | 5.05 | 7.2 | 4.93 | 7.1 | 5.17 | 7.3 |
| 15 | 5.29 | 7.4 | 5.17 | 7.3 | 5.05 | 7.2 |
| 20 | 5.29 | 7.4 | 5.23 | 7.4 | 5.05 | 7.2 |
| 25 | 5.41 | 7.6 | — | — | — | — |

tissue, the pH became more alkaline in tumour 2 but the results were equivocal in the other tumours. The tumour cell line, A15A5, used in this study has been characterized both *in vitro* and *in vivo* with both optical and electron microscopy (Claisse *et al.*, 1979; Davaki & Lantos, 1980, 1981). The cells of this line give rise to malignant astrocytomas when injected s.c. or intracerebrally into appropriate hosts (Lantos *et al.*, 1976; Davaki & Lantos, 1981).

The histology of the tumours depended to some extent on the passage number. The 4 tumours transplanted after 23 passages consisted mainly of two cell types. The first was a pleomorphic cell, polygonal, stellate or bipolar with oval or round hyperchromatic nuclei and abundant eosinophilic cytoplasm. These cells were frequently seen around blood vessels and also found in the tumours which had been injected after 34 passages. The second type of cell present mainly in the periphery, was spindle shaped and formed a loosely woven tissue in which the cells were haphazardly distributed in the fibrillary matrix.

A third type of cell was unique to the tumours injected after 34 passages. This cell was bipolar with elongated nuclei which contained a finely stippled chromatin. The Feulgen's reaction was less evident in these cells and they also showed somewhat less affinity for gallocyanin. These cells formed tightly packed parallel or interlacing bundles and appeared to be mitotically active.

The trichrome stain revealed collagen in all the tumours. This was more evident at the periphery of the tumour and was most prominent in areas composed of spindle shaped cells. All the tumours showed evidence of necrosis, the severity of which was related to the size of the neoplasm. The extent of necrosis, however, varied within the same tumour: small serpiginous and massive confluent necrosis was seen, and the latter frequently became cystic.

The vascularity of the tumours was variable, but even in the best vascularized areas the pronounced endothelial hyperplasia, associated with malignant gliomas, did not occur. Binucleate cells were occasionally seen but multinucleate giant cells were absent. Staining of astrocytic fibres with PTAH gave equivocal results. The reaction to glial fibrillary astrocytic protein (GFAP) was entirely negative.

On the whole the first four neoplasms (passage number 23) were smaller, better vascularized and had undergone less devastating necrosis than the second group of neoplasms (passage number 34). These features and the greater mitotic activity of the cells in the second group all suggest that tumours produced by cells of higher passage number are more malignant.

The first 4 animals had both intracranial and extracranial tumours. The latter were the result of the tumour growing back along the injection site through the small cranial burr hole. The intracranial neoplasms showed far less variety in cell type and cellular arrangements, the cells resembling the first type described above. The nuclei of all the tumour cells showed a much more marked Feulgen's reaction and affinity for gallocyanin than the surrounding cells of the cerebral tissue.

### Discussion

Atkinson (1977) believed that for most cells under nearly all conditions of steady state metabolism, the adenylate energy charge has a value between 0.87 and 0.94. Lowry *et al.* (1977) directly measured the high energy phosphate content of biopsy material from human gliomas and found values of adenylate charge between 0.57 to 0.91. In our study there are so many assumptions underlying the conversion of the [ADP]/[ATP] ratio to adenylate energy charge that the values found are likely to be only rough approximations. For example, one of the assumptions is that the pH environment within and between tumours is constant and this is clearly not the case as illustrated in Tables I and II. $[Mg]^{++}$ will also affect the value of the energy charge and the value of this variable is completely unknown in our study (Gupta & Yushok, 1980; Hoult *et al.*, 1974). Furthermore the meaning and role of the adenylate energy charge concept for both *in vitro* and *in vivo* systems has been criticized by Purich & Fromm (1973). It should also be pointed out that in several tissues the concentrations of ADP and Pi measured by *in vivo* $^{31}$P NMR are consistently lower than measurements made by more conventional methods (Ackerman *et al.*, 1980; Iles *et al.*, 1982). Nevertheless the 3 tumours which showed zero or near zero ratios of [PCr]/[ATP] because of an absent PCr peak on the NMR scan were the 3 tumours with the lowest adenylate energy charge. These three values are outside the range given by Atkinson (1977). Associated with this low [PCr]/[ATP] ratio and adenylate energy charge was an increased [SP]/[ATP] and [Pi]/[ATP] ratio both as a result of great increases in [SP] and [Pi]. Further evidence to suggest that the low adenylate energy charge was not the result of a lack of glucose substrate comes from the failure of an i.p. glucose infusion to stimulate the production of PCr.

All 3 tumours with the low energy charge belonged to the group of tumours produced by cells at 34 passages. These tumours were less differentiated than the tumours with 23 passages. A third type of cell with diminished RNA and DNA was evident in all of these more malignant tumours, including the 2 tumours with "normal" adenylate energy charge. There were no other factors such as pH, degree of necrosis or tumour size that could be exclusively associated with the fall in adenylate energy charge.

The tumours used in these experiments had greatly differing growth rates and presumably different metabolic patterns. The change in the high energy phosphate metabolism noted in the second, more malignant group of tumours may be related to the appearance of a new cell type. Only serial studies will reveal if the reduced adenylate charge associated with diminished PCr occurs in the early stages of growth or if it is a feature of late growth.

We wish to acknowledge the assistance of Mr. B. Deane who transplanted the tumours, Mr. P. Martin who performed much of the NMR spectroscopy, Dr. C. Scholtz who arranged for the histological stains and the MRC Biomedical NMR Centre, NIMR, Mill Hill for making NMR facilities available.

**References**

ACKERMAN, J.J.H., GROVE, T.H., WONG, G.G., GADIAN, D.G. & RADDA, G.K. (1980). Mapping of metabolites in whole animals by $^{31}$p NMR using surface coils. *Nature*, **283**, 167.

ATKINSON, D.E. (1977). *Cellular Energy Metabolism and its Regulation*. New York: Academic Press.

CLAISSE, P.J., ROSCOE, J.P. & LANTOS, P.L. (1979). Cellular heterogeneity in an ethylnitrosourea-induced glioma: Malignancy, karyology and other properties of tumour cell types. *Br. J. Exp. Pathol.*, **60**, 209.

DAVAKI, P. & LANTOS, P.L. (1980). Morphological analysis of malignancy: A comparative study of transplanted brain tumours. *Br. J. Exp. Pathol.*, **61**, 655.

DAVAKI, P. & LANTOS, P.L. (1981). The development of brain tumours produced in rats by the intracerebral injection of neoplastic glial cells: A fine structural study. *Neuropathol. Appl. Neurobiol.*, **7**, 49.

GADIAN, D.G. (1977). Nuclear magnetic resonance in living tissue. *Contemp. Phys.*, **18**, 351.

GADIAN, D.G. (1982). *NMR and its Application to Living Systems*. Oxford: Oxford University Press.

GORDON, R.E., HANLEY, P.E. & SHAW, D. (1982). Topical magnetic resonance. *Prog. NMR Spectrosc.*, **15**, 1.

GRIFFITHS, J.R. & ILES, R.A. (1980). Nuclear magnetic resonance—A "magnetic eye" on metabolism. *Clin. Sci.*, **59**, 225.

GRIFFITHS, J.R., STEVENS, A.N., ILES, R.A., GORDON, R.E. & SHAW, D. (1981). $^{31}$P NMR investigations of solid tumours in the living rat. *Biosci. Rep.*, **1**, 319.

GUPTA, R.K. & YUSHOK, W.D. (1980). Noninvasive $^{31}$P NMR probes of free Mg, MgATP and MgADP in intact Ehrlich ascites tumor cells. *Proc. Natl Acad. Sci.*, **77**, 2487.

HOULT, D.I., BUSBY, S.J.W., GADIAN, D.G., RADDA, G.K., RICHARDS, R.E. & SEELY, P.J. (1974). Observations of tissue metabolites using $^{31}$P nuclear magnetic resonance. *Nature*, **252**, 285.

ILES, R.A., STEVENS, A.N. & GRIFFITHS, J.R. (1982). NMR studies of metabolites in living tissue. *Prog. NMR Spectrosc.*, **15**, 49.

KOEZE, T.H. (1982). Applications of nuclear magnetic resonance in medicine. *Br. J. Hosp. Med.*, **27**, 402.

KOEZE, T.H. (unpublished observations).

LANTOS, P.L., ROSCOE, J.P. & SKIDMORE, C.J. (1976). Studies of the morphology and tumourigenicity of experimental brain tumours in tissue culture. *Br. J. Exp. Pathol.*, **57**, 95.

LOWRY, O.H., BERGER, S.J., CHI, M.M.-Y., CARTER, J.G., BLACKSHAW, A. & OUTLAW, B. (1977). Diversity of metabolic patterns in human brain tumours—I. High energy phosphate compounds and basic composition. *J. Neurochem.*, **29**, 959.

NAVON, G., OGAWA, S., SHULMAN, R.G. & YAMANE, T. (1977). $^{31}$P Nuclear magnetic resonance studies of Ehrlich ascites tumor cells. *Proc. Natl Acad. Sci.*, **74**, 87.

PURICH, D.L. & FROMM, H.J. (1973). Additional factors influencing enzyme responses to adenylate energy charge. *J. Biol. Chem.*, **25**, 461.

RACKER, E. (1976). Why do tumour cells have a high aerobic glycolysis. *J. Cell. Physiol.*, **89**, 697.